# Associations of pre-pandemic levels of physical function and physical activity with COVID-19-like symptoms during the outbreak

**DOI:** 10.1007/s40520-021-02006-7

**Published:** 2021-10-30

**Authors:** Marguerita Saadeh, Amaia Calderón-Larrañaga, Davide Liborio Vetrano, Philip von Rosen, Laura Fratiglioni, Anna-Karin Welmer

**Affiliations:** 1 Aging Research Center, Department of Neurobiology, Care Sciences and Society, Karolinska Institutet & Stockholm University, Solna, Sweden; 2 Centro di Medicina dell’Invecchiamento, 11 IRCCS Fondazione Policlinico “A. Gemelli” and Catholic University of Rome, Rome, Italy; 3 Division of Physiotherapy, Department of Neurobiology, Care Sciences and Society, Karolinska Institutet, Stockholm, Sweden; 4 Stockholm Gerontology Research Center, Stockholm, Sweden; 5 Women’s Health and Allied Health Professionals Theme, Medical Unit Medical Psychology, Karolinska University Hospital, Stockholm, Sweden

**Keywords:** Coronavirus disease 2019, Muscle strength, Mobility, Physical activity, Older adults

## Abstract

**Background:**

One’s physical function and physical activity levels can predispose or protect from the development of respiratory infections. We aimed to explore the associations between pre-pandemic levels of physical function and physical activity and the development of COVID-19-like symptoms in Swedish older adults.

**Methods:**

We analyzed data from 904 individuals aged ≥ 68 years from the population-based Swedish National study on Aging and Care in Kungsholmen. COVID-19-like symptoms were assessed by phone interview (March–June 2020) and included fever, cough, sore throat and/or a cold, headache, pain in muscles, legs and joints, loss of taste and/or odor, breathing difficulties, chest pain, gastrointestinal symptoms, and eye inflammation. Muscle strength, mobility, and physical activity were examined in 2016–2018 by objective testing. Data were analyzed using logistic regression models in the total sample and stratifying by age.

**Results:**

During the first outbreak of the pandemic, 325 (36%) individuals from our sample developed COVID-19-like symptoms. Those with slower performance in the chair stand test had an odds ratio (OR) of 1.5 (95% confidence interval [CI] 1.1–2.1) for presenting with COVID-19-like symptoms compared to better performers, after adjusting for potential confounders. The association was even higher among people aged ≥ 80 years (OR 2.6; 95% CI 1.5–4.7). No significant associations were found between walking speed or engagement in moderate-to-vigorous physical activity and the likelihood to develop COVID-19-like symptoms.

**Conclusion:**

Poor muscle strength, a possible indicator of frailty, may predispose older adults to higher odds of developing COVID-19-like symptoms, especially among the oldest-old.

## Introduction

The SARS-CoV-2 pandemic has spread globally impacting health and the economy on an unprecedented scale [1]. In Sweden, as well as in other countries, the public health agency recommended limiting the number of social interactions and ensuring that those with even slight symptoms refrained from meeting others, and older adults were advised to follow stricter isolation recommendations [2].

SARS-CoV-2 infection often goes unrecognized, especially if of mild severity. This might have been particularly true right after the first outbreak in spring 2020, when contact tracing and testing were on their way to being fully implemented in Sweden. COVID-19-like symptoms may be an outward sign of the illness, and can serve as a proxy for a test when testing is not available [1, 3]. Commonly reported symptoms include fever, cough, sore throat, headache, pain in muscles, legs and joints, loss of taste and/or odor, breathing difficulties, chest pain, gastrointestinal symptoms (diarrhea, nausea, and vomiting), and eye inflammation [1, 4].

Physical function, including muscle strength and mobility, has been identified as a good indicator of healthy aging for its capability to predict future health status and mortality [5]. Muscle strength may serve as a proxy of muscle quality/health and is considered essential for the body's movement, facilitates respiratory system functions, and is important for proper immune response [6, 7]. Moreover, walking speed, considered the sixth vital sign, is a good measure of mobility and a powerful marker of cardiopulmonary function and represents a global measure of physical function, reserve, and resilience [8]. Also, the protective effect of physical activity on physical frailty (i.e., characterized by diminished strength, resilience, and physiologic function) and cardiovascular diseases has been extensively demonstrated [9, 10]. Furthermore, physical activity may help strengthening and maintaining the immune system making the individual less susceptible to infections [11]. As COVID-19 affects primarily the cardiopulmonary system; physical function and physical activity are key targets for investigation with important implications for future COVID-19—as well as other respiratory infections—research and public health recommendations.

Despite the growing amount of literature on the risk factors for developing COVID-19-like symptoms, so far, its association with previous levels of physical function and physical activity remains unclear. The aim of our study was to explore the association between muscle strength, mobility, physical activity, and the likelihood of developing COVID-19-like symptoms in Swedish older adults.

## Methods

### Study population

Our study population is from the sixth wave follow-up (2016–2018) of the Swedish National Study on Aging and Care in Kungsholmen (SNAC-K) (www.snac-k.se). SNAC-K is a longitudinal study including a random sample of older adults aged 60 years and above living in the Kungsholmen district of Stockholm. At baseline (2001–2003), 3363 individuals (73.3% participation rate) were selected from 11 age cohorts (ages 60, 66, 72, 78, 81, 84, 87, 90, 93, 96, and ≥ 99 years) and have been followed up regularly: every 6 years for the young-old cohorts (< 78 years) and every 3 years for the older cohorts (≥ 78 years). Three additional cohorts were added in 2007–2009 (81 years old), 2010–2012 (60 years old), and 2013–2015 (81 years old). The sixth wave follow-up included participants from age groups 66, 81, 84, 87, 90, 93, and ≥ 96 years, who underwent extensive clinical examinations, interviews, and assessments by physicians, nurses, and psychologists following the same protocols as in all study waves. All participants from the sixth wave were invited to participate in a telephone interview between May and September 2020 (95% of the interviews were conducted in May and June). Information on their physical and mental health and psychosocial conditions during the COVID-19 pandemic were obtained by trained nurses, following standard protocols. Individuals with severely impaired hearing, diagnosed with dementia or living in nursing homes were excluded from the study. The response rate was 91.9%. The telephone interview was carried out after COVID-19 was declared a pandemic [12].

Out of 1231 individuals that participated in the telephone interview, 950 also participated in wave 6 (2016–2018). We further excluded 46 (4.8%) individuals with missing information on the chair stand or walking speed tests during the sixth wave follow-up. Thus, our analytical sample for chair stands and walking speed consisted of 904 participants (95.2% of the phone interview sample). Concerning physical activity, a sub-sample of 587 subjects (47.7% of the phone interview sample) had data in wave 6. Participants eligible to wear the activPAL3 accelerometer were free from severe cognitive impairment or indoor mobility limitation and agreed to wear the activPAL3 for seven consecutive days. Accelerometer data of participants with at least four valid measurement days (i.e., if wear time was of at least 10 h during waking hours) were included in the analyses [13].

SNAC-K was approved by the Regional Ethical Review Board in Stockholm, and written informed consent was obtained from participants or their next of kin. The SNAC-K COVID-19 study was also approved by the Regional Ethical Review Board in Stockholm (dnr: 2020-02497).

### Muscle strength and mobility

The chair stand test was performed by asking participants to fold their arms across their chest and stand up from a seated position five times consecutively as quickly as possible, and the results were expressed in seconds. Walking speed was assessed over 6 m or, if the participant reported walking slowly, 2.4 m at a self-selected speed. It was reported as meters per second (m/s), reflecting the time for whichever length walked. Participants who were unable to perform any of the lower extremity tests due to severe physical limitations received the worst possible score; that is, a 75-s chair stand time or, a walking speed of 0 m/s [14]. Poor muscle strength was defined as a chair stand time ≥ 11 s according to the median of the distribution, and mobility limitation was defined as a walking speed < 0.8 m/s, as previously suggested [15].

### Moderate-to-vigorous physical activity

The activPAL3 accelerometer (PAL Technologies Ltd., Glasgow, UK) was used to assess physical activity during the sixth wave follow-up [13]. The activPAL3 is a small and slim thigh-worn activity monitor that uses triaxial acceleration to determine thigh angle and body posture (i.e., sitting/lying or upright), along with transitions between these postures and stepping speed (cadence). The activPAL3 has high accuracy for measuring time spent sedentary, standing or stepping, and for speed of movement [16]. Participants were asked to continue with their usual physical activity habits while wearing the activPAL3 for seven consecutive days during all waking hours (excluding water-based activities) starting the day after the interview, and to record the time when they put on and remove the device each day on a log sheet. In this study, physical inactivity was defined as performing moderate-to-vigorous physical activity (MVPA) < 150 min/week, in accordance with current guidelines [17]. Previous research has shown that 100 steps/min is an appropriate threshold value corresponding to three metabolic equivalents (METs), which is analogous to MVPA levels. Consequently, we used a cadence of ≥ 100 steps/min to define MVPA [18].

### COVID-19-like symptoms

Testing for COVID-19 was still not widespread in Sweden at the time of the first outbreak of the pandemic and during the telephone interview; therefore, it was not possible to determine who was actually infected by the SARS-CoV-2 virus. In a study by Adorni et al. [3], the authors concluded that self-reported symptoms are reliable indicators of SARS-CoV-2 infection in a pandemic context and having at least one COVID-19-like symptom was shown to be associated with an increased likelihood of having COVID-19. Therefore, in this study, we used COVID-19-like symptoms at the time of the first outbreak as a proxy to determine who might have been potentially infected. During the telephone interview, participants were asked if, since march 2020, they had experienced any of the following symptoms (yes/no): fever (> 37.5°C for three consecutive days), cough, sore throat and/or a cold, headache, pain in muscles, legs and joints, loss of taste and/or odor, breathing difficulties, chest pain, gastrointestinal symptoms (diarrhea, nausea, and vomiting), and eye inflammation. The presence of COVID-19-like symptoms was analyzed both as continuous and dichotomous (i.e., zero vs at least one symptom and zero/one vs at least two symptoms) variables.

### Covariates

Several covariates were considered as possible confounders: age (continuous), sex (male or female), highest level of education (elementary school/high school or university and above), living alone (yes or no), chronic obstructive pulmonary disease (COPD) (yes or no), asthma (yes or no), other respiratory diseases (yes or no), number of chronic cardiovascular, neuropsychiatric or musculoskeletal diseases (continuous) [19], all assessed in wave 6 (2016–2018); as well as location where most time was spent since March 2020 (at home/with a relative or at senior, service, nursing housing/hospital/rehabilitation), frequency of going out since March 2020 (every day or ≥ 1 times per week or < 1 times per week/never), and use of public transport since March 2020 (≥ 1 per week or 2–3 times per month or < 2 times per month/never), all assessed during the phone interview (May–September 2020).

### Statistical analysis

Characteristics of the participants by age were compared using the Chi-square test. Logistic regressions were employed to estimate the odds ratios (OR) and 95% confidence intervals (CI) for the association between muscle strength, mobility, and physical activity levels and COVID-19-like symptoms. Models were first adjusted for sex, age, and education level (Model I), and additionally for living alone, location where most time was spent during the pandemic, frequency of going out, use of public transport, and number of chronic cardiovascular, neuropsychiatric, and musculoskeletal diseases (Model II). All exposures were dichotomized according to the median of the distribution (i.e., chair stands) or clinical/recommended cut-offs (i.e., walking speed and MVPA) to address potential non-linearity in their association with the outcome, and to facilitate the interpretation of the findings. The presence of statistical interactions between the exposures (i.e., chair stand time, walking speed, and MVPA) and age (< 70 vs. ≥ 80 years) were examined. We then performed stratified analyses by age groups. As sensitivity analyses, we reran the models after: (a) mutually adjusting by chair stand time, walking speed, and physical activity in the sub-sample of *n* = 587; (b) using linear regression with the number of COVID-19-like symptoms as the outcome, to check for potential linearity of associations in the study sample (*N* = 904); and (c) dichotomizing the number of COVID-19-like symptoms as < 2 or ≥ 2 in the study sample (*N* = 904). All analyses were performed using Stata version 15 with the level of statistical significance set at *p* < 0.05.

## Results

**Fig. 1:**
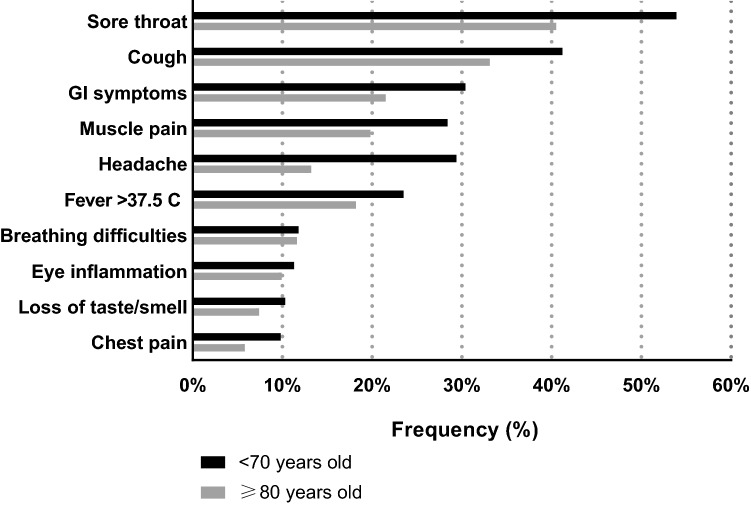
Frequency of COVID-19-like symptoms among the 325 individuals reporting at least one symptom by age groups. *GI symptoms* gastrointestinal symptoms

The study population consisted of 904 individuals, 64.9% being female, with a mean age of 77.9 (standard deviation [SD] 9.3) years, of whom 49.9% had poor muscle strength and 16.2% mobility limitation (Table 1). Among individuals older than 80 years, 73.3% had poor muscle strength and 29.8% had mobility limitation (Table 1). Most participants did not report any COVID-19-like symptom (64.1%) during the survey period, while 36% and 21% reported having at least one or at least two COVID-19-like symptoms, respectively (Table 1). Only 46 participants (5% of the total analytical sample) were tested for COVID-19 infection, and 8 of them were positive (17.4% of those who had done the test) and reported having at least one COVID-19-like symptom. Similar characteristics were seen for the sub-sample for which physical activity was measured, which consisted of 587 individuals with a mean (SD) age of 76 (8.6) years, of whom 38.3% were physically inactive (62.5% of those older than 80 years) (Additional Table a3). Also, those that reported having at least one COVID-19-like symptom were less healthy, had mobility limitation, poorer muscle strength, and were physically inactive (Additional Tables a4-a5). Individuals older than 80 years had a lower prevalence of COVID-19-like symptoms compared to those younger than 70 years (Fig. 1). The most common COVID-19-like symptoms among participants with at least one symptom were having sore throat, cough, gastrointestinal symptoms, and muscle pain (Fig. 1).

**Table 1:** Sociodemographic, clinical, and lifestyle characteristics of the study sample by age groups (*N* = 904)

	Total population (*N* = 904)	< 70 years (*n* = 475)	≥ 80 years (*n* = 429)	*p* value*
*Variables assessed at wave 6 (2016–2018)*				
Female, *n* (%)	587 (64.9)	288 (60.6)	299 (69.7)	**0.004**
Education, *n* (%)				
Elementary/high school	382 (42.3)	152 (32.0)	230 (53.6)	** < 0.001**
University	522 (57.7)	323 (68.0)	199 (46.4)	
≥ 1 cardiovascular disease, *n* (%)	244 (27.0)	61 (12.8)	183 (42.7)	** < 0.001**
≥ 1 neuropsychiatric disease, *n* (%)	229 (25.3)	126 (26.5)	103 (24.0)	0.385
≥ 1 musculoskeletal disease, *n* (%)	538 (59.5)	218 (45.9)	320 (74.6)	** < 0.001**
COPD, *n* (%)	50 (5.5)	14 (3.0)	36 (8.4)	** < 0.001**
Asthma, *n* (%)	85 (9.4)	36 (7.6)	49 (11.4)	**0.048**
Other respiratory diseases, *n* (%)	11 (1.2)	6 (1.3)	5 (1.2)	0.894
Poor muscle strength, *n* (%)	451 (49.9)	137 (28.8)	314 (73.2)	** < 0.001**
Mobility limitation, *n* (%)	146 (16.2)	18 (3.8)	128 (29.8)	** < 0.001**
*Variables assessed during the telephone interview (May–September 2020)*				
Living alone, *n* (%)	452 (50)	174 (36.6)	278 (64.8)	** < 0.001**
Location where most time spent (since March), *n* (%)				
Home/with relatives	858 (96.5)	462 (99.4)	396 (93.4)	** < 0.001**
Senior, service, nursing housing/hospital/rehab	31 (3.5)	3 (0.7)	28 (6.6)	
Frequency of going out (since March), *n* (%)				
Everyday	683 (76.3)	415 (87.9)	268 (63.4)	** < 0.001**
≥ 1 per week	151 (16.9)	49 (10.4)	102 (24.1)	
< 1 per week/never	61 (6.8)	8 (1.7)	53 (12.5)	
Use of public transport (since March), *n* (%)				
≥ 1 per week	122 (13.7)	78 (16.5)	44 (10.5)	**0.031**
2–3 times per month	88 (9.8)	44 (9.3)	44 (10.5)	
< 2 times per month/never	684 (76.5)	351 (74.2)	333 (79.1)	
≥ 1 COVID-19-like symptoms, *n* (%)	325 (35.9)	204 (43.0)	121 (28.2)	** < 0.001**
≥ 2 COVID-19-like symptoms, *n* (%)	188 (20.8)	132 (27.8)	56 (13.1)	** < 0.001**

Poor muscle strength defined as a chair stand test time below the median of the distribution (i.e., ≥ 11 s). Mobility limitation defined according to a previously suggested clinical cut-off for walking speed (i.e., < 0.8 m/s)

*COPD* chronic obstructive pulmonary disease

*Chi^2^ test

GI symptoms gastrointestinal symptoms.

**Fig. 2:**
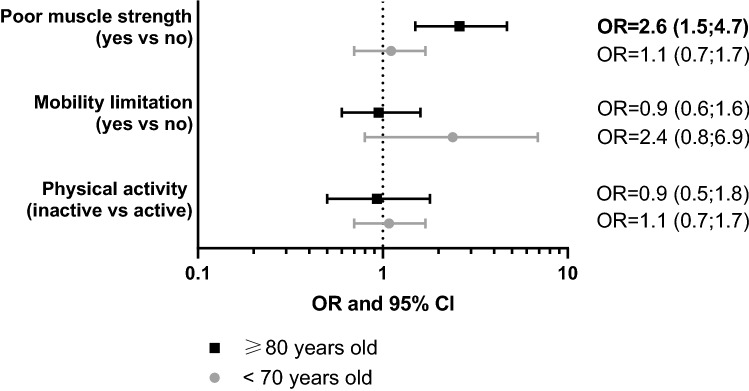
Associations of poor muscle strength, mobility limitation, and physical activity with presence of COVID-19-like symptoms (0 vs 1 +). Results derived from three separate logistic regression models for each of the two age groups

Individuals with poor muscle strength had 50% higher odds (OR = 1.5; 95% CI 1.1–2.1) of presenting with at least one COVID-19-like symptom compared to those with normal muscle strength, after adjusting for potential confounders. The associations with COVID-19-like symptoms were non-significant for mobility limitation and physical activity (Table 2). We found significant interactions of muscle strength with age (≥ 80 years vs < 70 years) in relation to the outcome (*p*_interaction_ = 0.045) (Fig. 2). In fact, individuals older than 80 years with poor muscle strength had 160% higher odds (OR = 2.6; 95% CI 1.5–4.7) of presenting with at least one COVID-19-like symptom compared to those with normal muscle strength. There was a tendency for individuals younger than 70 years with mobility limitation to have higher odds of presenting with COVID-19-like symptoms compared to those with better mobility; however, neither the association nor the interaction with age were statistically significant (*p*_interaction_ = 0.065) (Fig. 2). Results remained the same after mutually adjusting by all exposures (Additional Table a6). When analyzing the outcome as a continuous variable and dichotomized as having 0–1 vs. ≥ 2 COVID-19-like symptoms, an association with poor muscle strength was still detected, but this was statistically significant only among subjects older than 80 years (Additional Tables a7-a8).

**Table 2:** Associations of poor muscle strength, mobility limitation, and physical activity levels with presence of COVID-19-like symptoms (0 vs 1 +). Results from logistic regression models

	Model I		Model II	
	OR (95% CI)	*p* value	OR (95% CI)	*p* value
Poor muscle strength (*N* = 904)				
No	Ref.	Ref.	Ref.	Ref.
Yes	**1.55 (1.1–2.1)**	**0.007**	**1.50 (1.1–2.1)**	**0.017**
Mobility limitation (*N* = 904)				
No	Ref.	Ref.	Ref.	Ref.
Yes	1.30 (0.9–2.0)	0.225	1.11 (0.7–1.7)	0.721
Physical activity (*n* = 587)				
Active	Ref.	Ref.	Ref.	Ref.
Inactive	1.10 (0.7–1.6)	0.631	1.04 (0.7–1.6)	0.848

Model I: adjusted by age, sex, and education level. Model II: adjusted additionally by living alone, location where most time spent, frequency of going out, use of public transport, number of chronic cardiovascular, neuropsychiatric, and musculoskeletal diseases, COPD, asthma, and other respiratory diseases. Poor muscle strength defined as a chair stand test time below the median of the distribution (i.e., ≥ 11 s). Mobility limitation defined according to a previously suggested clinical cut-off for walking speed (i.e., < 0.8 m/s). Physical inactivity defined according to current guidelines’ definition of moderate-to-vigorous physical activity levels (i.e., < 150 min/week)

Models adjusted by age, sex, education level, living alone, location where most time spent, frequency of going out, use of public transport, number of chronic cardiovascular, neuropsychiatric, and musculoskeletal diseases, COPD, asthma, and other respiratory diseases. Interaction with age: poor muscle strength (yes vs no) # age (≥ 80 years vs < 70 years): OR = 2.04; *p* = 0.045. Mobility limitation (yes vs no) # age (≥ 80 years vs < 70 years): OR = 0.34; *p* = 0.065. Physical activity (inactive vs active) # age (≥ 80 years vs < 70 years): OR = 0.96; *p* = 0.913. Poor muscle strength defined as a chair stand test time below the median of the distribution (i.e., ≥ 11 s). Mobility limitation defined according to a previously suggested clinical cut-off for walking speed (i.e., < 0.8 m/s). Physical inactivity defined according to current guidelines’ definition of moderate-to-vigorous physical activity levels (i.e., < 150 min/week). The association of physical activity and COVID-19-like symptoms was performed in the sub-sample of 587 participants.

## Discussion

Using data from a Swedish population-based study of older adults aged 68 years and above, we found that individuals with poor muscle strength had a higher risk of developing COVID-19-like symptoms, especially among the oldest-old adults, after adjusting for potential confounders.

Most literature on risk factors related to COVID-19 incidence and severity has focused on aspects of health other than physical function, such as hospitalization, individual chronic conditions, or multimorbidity [20–23]. Similarly to our results, a study of 3241 confirmed cases of COVID-19-related deaths showed that individuals older than 90 years had less COVID-19-related symptoms compared to those younger than 60 years [23]. Recent evidence on the association between physical function and COVID-19 supports our finding that poor muscle strength, as a possible indicator of frailty, may also be an important risk factor [24]. Notably, two recent systematic reviews concluded that frail older adults diagnosed with COVID-19 are at a higher risk of severe complications and mortality [25, 26]. For instance, a study conducted in ten hospitals in the UK and one in Italy concluded that frail patients were more likely to die from COVID-19 and stay longer in the hospital than those who were not frail [27]. Similarly, a study on 3,600 adults aged 50 years and above living in 27 European countries showed that those with better muscle strength, measured through grip strength, had a lower risk of hospitalization due to COVID-19 [28].

We did not find a significant association between mobility or physical activity and COVID-19-like symptoms, although there was an indication of an association with mobility limitation among the younger participants. In line with this indication, a study including 414,201 UK Biobank participants showed that those with mobility limitation had the highest risk of developing severe COVID-19 symptoms [29]. Previous studies have also shown that cardiovascular fitness and aerobic exercise (e.g., walking) induce a proper cardiorespiratory function and a better immune system [30]. Indeed, physical fitness and levels of aerobic exercise are important correlates of reduced frequency of upper respiratory tract infection and severity of symptoms [11]. Furthermore, previous research has shown that physical activity interventions can delay or prevent frailty progression and age-related decline in immune response [31]. However, past studies showing significant associations between physical activity/aerobic exercise and an improved immune system mainly included younger individuals [32]. The few studies that examined these associations in older populations suggest little or no effect of aerobic exercise on the immune system [33, 34].

Poor muscle strength, as a proxy of muscle quality/health, may also be a marker of impairment in several body systems and organs that could potentially explain the associations we found with COVID-19-like symptoms. Indeed, poor muscle strength has been shown to be related to the immune, cardiovascular, and respiratory systems [7, 8, 35], which play an important role in providing older adults with greater resilience against infections and the development of severe symptoms. Previous studies have found that various aspects related to aging, including frailty and chronic inflammation, can impair the immune response [7, 27, 36], which may partially explain the higher risk of severe symptoms and mortality from COVID-19 among older adults. Also, the age-related deterioration of the immune system, the so-called immunosenescence, which is characterized, among other aspects, by an increase in the number of dysfunctional immune T cells, contributes to an increased incidence of infections and poorer vaccine response [37]. It has been suggested that better muscle health and higher levels of strength training may partially counteract the age-related increase in senescent T cells [38], which could be one explanation to our finding of an association between poor muscle strength and the presence of COVID-19-like symptoms, especially among the oldest-old.

Strengths of this study include its longitudinal design and large sample of older adults with detailed functional and clinical characterization and available data on several potential confounders, such as chronic conditions or behavioral factors. Moreover, we used objective measures of chair stand time, walking speed and MVPA assessed by qualified healthcare professionals and accelerometers, reducing the risk of recall or report bias from self-reported data. However, some limitations need to be acknowledged. The study sample included participants aged < 70 and ≥ 80 years, that are healthy and relatively wealthy and able to self-report their behaviors and COVID-19-like symptoms. Thus, generalization of our results to other age groups of older adults or even to the general population should be done with caution. In addition, since data on COVID-19 were collected through telephone interviews, those who died because of COVID-19 or were less healthy at baseline were excluded from the study. This might have led to an underestimation of the association under study, as those individuals most likely had poorer physical function and worse COVID-19 health outcomes. Recall bias might not be completely avoided due to self-reported COVID-19-like symptoms, especially among participants presenting with neuropsychiatric diseases. Despite adjusting our models for several confounders, residual confounding may not be fully discarded. Finally, as testing for COVID-19 was still not widespread in Sweden at the time of the telephone interview, it is not possible to determine who was actually infected by the SARS-CoV-2 virus.

## Conclusion

As countries started to implement COVID-19 vaccine strategies to prevent people from becoming severely ill, identifying individuals at greatest risk of developing symptoms is crucial. This study highlights the importance of physical function and, more specifically, muscle strength as a potential risk factor for developing COVID-19-like symptoms. Further longitudinal studies are needed to confirm our results and to better understand the mechanisms through which muscle strength is associated with a higher risk of developing COVID-19-like symptoms, as an important step to design successful holistic interventions to make people more resilient to the infection.

## Data Availability

Data are from the SNAC-K project, a population-based study on aging and dementia (http://www.snac-k.se/). Access to these original data is available to the research community upon approval by the SNAC-K data management and maintenance committee. Applications for accessing these data can be submitted to Maria Wahlberg (Maria.Wahlberg@ki.se) at the Aging Research Center, Karolinska Institutet.

## Appendix

See Tables 3, 4, 5, 6, 7, 8.

**Table 3:** Sociodemographic, clinical, and lifestyle characteristics of the physical activity assessment sub-sample by age groups (*n* = 587)

	Total population (*N* = 587)	< 70 years (*n* = 358)	≥ 80 years (*n* = 229)	*p* value*
*Variables assessed at wave 6 (2016–2018)*				
Female, *n* (%)	380 (64.7)	219 (61.2)	161 (70.3)	**0.024**
Education, *n* (%)				
Elementary/high school	245 (41.7)	117 (32.7)	128 (55.9)	** < 0.001**
University	342 (58.3)	241 (67.3)	101 (44.1)	
≥ 1 cardiovascular disease, *n* (%)	129 (22.0)	44 (12.3)	85 (37.1)	** < 0.001**
≥ 1 neuropsychiatric disease, *n* (%)	127 (21.6)	79 (22.1)	48 (21.0)	0.751
≥ 1 musculoskeletal disease, *n* (%)	331 (56.4)	164 (45.8)	167 (72.9)	** < 0.001**
COPD, *n* (%)	29 (4.9)	8 (2.2)	21 (9.2)	** < 0.001**
Asthma, *n* (%)	57 (9.7)	25 (7.0)	32 (14.0)	**0.005**
Other respiratory diseases, *n* (%)	7 (1.2)	4 (1.1)	3 (1.3)	0.834
Physical inactivity, *n* (%)	225 (38.3)	82 (22.9)	143 (62.5)	** < 0.001**
*Variables assessed during the telephone interview (May–September 2020)*				
Living alone, *n* (%)	272 (46.3)	123 (34.4)	149 (65.1)	** < 0.001**
Location where most time spent (since March), *n* (%)				
Home/with relatives	563 (97.6)	350 (99.7)	213 (94.3)	** < 0.001**
Senior, service, nursing housing/hospital/rehab	14 (2.4)	1 (0.3)	13 (5.8)	
Frequency of going out (since March), *n* (%)				
Everyday	481 (82.5)	316 (89.0)	165 (72.4)	** < 0.001**
≥ 1 per week	77 (13.2)	34 (9.6)	43 (18.9)	
< 1 per week/ never	25 (4.3)	5 (1.4)	20 (8.8)	
Use of public transport (since March), *n* (%)				
≥ 1 per week	76 (13.0)	52 (14.6)	24 (10.6)	0.370
2–3 times per month	60 (10.3)	36 (10.1)	24 (10.6)	
< 2 times per month/never	447 (76.7)	268 (75.3)	179 (78.9)	
≥ 1COVID-19-like symptoms, *n* (%)	207 (35.3)	144 (40.2)	63 (27.5)	** < 0.001**
≥ 2 COVID-19-like symptoms, *n* (%)	119 (20.3)	89 (24.9)	30 (13.1)	** < 0.001**

Physical inactivity defined according to current guidelines’ definition of moderate-to-vigorous physical activity levels (i.e., < 150 min/week)

*COPD* chronic obstructive pulmonary disease

*Chi-square test

**Table 4:** Sociodemographic, clinical, and lifestyle characteristics of the study sample by COVID-19 like symptoms (*N* = 904)

	< 70 years		*p* value*	≥ 80 years		*p *value*
	0 symptoms (*n* = 271)	≥ 1 symptoms (*n* = 204)		0 symptoms (*n* = 308)	≥ 1 symptoms (*n* = 121)	
*Variables assessed at wave 6 (2016–2018)*						
Female, *n* (%)	158 (58.3)	130 (63.7)	0.231	216 (70.1)	83 (68.6)	0.756
Education, *n* (%)						
Elementary/high school	93 (34.3)	59 (28.9)	0.212	163 (52.9)	67 (55.4)	0.647
University	178 (65.7)	145 (71.1)		145 (47.1)	54 (44.6)	
≥ 1 cardiovascular disease, *n* (%)	34 (12.6)	27 (13.2)	0.824	128 (41.6)	55 (45.6)	0.463
≥ 1 neuropsychiatric disease, *n* (%)	65 (24.0)	61 (29.9)	0.148	74 (24.0)	29 (24.0)	0.990
≥ 1 musculoskeletal disease, *n* (%)	120 (44.3)	98 (48.0)	0.416	222 (72.1)	98 (81.0)	**0.056**
COPD, *n* (%)	6 (2.2)	8 (3.9)	0.276	22 (7.1)	14 (11.6)	0.137
Asthma, *n* (%)	21 (7.8)	15 (7.4)	0.872	34 (11.0)	15 (12.4)	0.691
Other respiratory diseases, *n* (%)	3 (1.1)	3 (1.5)	0.725	4 (1.3)	1 (0.8)	0.682
Poor muscle strength, *n* (%)	74 (27.3)	63 (30.9)	0.394	213 (69.2)	101 (83.5)	**0.003**
Mobility limitation, *n* (%)	6 (2.2)	12 (5.9)	**0.038**	92 (29.9)	36 (29.8)	0.981
*Variables assessed during the telephone interview (May–September 2020)*						
Living alone, *n* (%)	90 (33.2)	84 (41.2)	**0.074**	200 (64.8)	78 (64.5)	0.927
Location where most time spent (since March), *n* (%)						
Home/with relatives	264 (99.2)	198 (99)	0.406	285 (93.8)	111 (92.5)	0.641
Senior, service, nursing housing/hospital/rehab	1 (0.4)	2 (1.0)		19 (6.3)	9 (7.5)	
Frequency of going out (since March), *n* (%)						
Everyday	240 (88.9)	175 (86.6)	0.491	199 (65.9)	69 (57.0)	0.164
≥ 1 per week	27 (10.0)	22 (10.9)		70 (23.2)	32 (26.5)	
< 1 per week/ never	3 (1.1)	5 (2.5)		33 (10.9)	20 (16.5)	
Use of public transport (since March), *n* (%)						
≥ 1 per week	35 (13.0)	43 (21.2)	**0.054**	34 (11.3)	10 (8.3)	0.131
2–3 times per month	25 (9.3)	19 (9.4)		26 (8.7)	18 (14.9)	
< 2 times per month/never	210 (77.8)	141 (69.5)		240 (80.0)	93 (76.9)	

Poor muscle strength defined as a chair stand test time below the median of the distribution (i.e., ≥ 11 s). Mobility limitation defined according to a previously suggested clinical cut-off for walking speed (i.e., < 0.8 m/s)

*COPD* chronic obstructive pulmonary disease

*Chi^2^ test

**Table 5:** Sociodemographic, clinical, and lifestyle characteristics of the physical activity assessment sub-sample by COVID-19 like symptoms (*N* = 587)

	< 70 years		*p* value*	≥ 80 years		*p* value*
	0 symptoms (*n* = 214)	≥ 1 symptoms (*n* = 144)		0 symptoms (*n* = 166)	≥ 1 symptoms (*n* = 63)	
*Variables assessed at wave 6 (2016–2018)*						
Female, *n* (%)	122 (57.0)	97 (67.4)	**0.049**	123 (74.1)	38 (60.3)	**0.042**
Education, *n* (%)						
Elementary/high school	76 (35.5)	41 (28.5)	0.164	87 (52.4)	41 (65.1)	0.085
University	138 (64.5)	103 (71.5)		79 (47.6)	22 (34.9)	
≥ 1 cardiovascular disease, *n* (%)	27 (12.6)	17 (11.8)	0.819	58 (34.9)	27 (42.9)	0.268
≥ 1 neuropsychiatric disease, *n* (%)	45 (21.0)	34 (23.6)	0.563	35 (21.1)	13 (20.6)	0.941
≥ 1 musculoskeletal disease, *n* (%)	90 (42.1)	74 (51.4)	0.082	118 (71.1)	49 (77.8)	0.309
COPD, *n* (%)	5 (2.3)	3 (2.1)	0.874	13 (7.8)	8 (12.7)	0.254
Asthma, *n* (%)	17 (7.9)	8 (5.6)	0.385	24 (14.5)	8 (12.7)	0.732
Other respiratory diseases, *n* (%)	2 (0.9)	2 (1.4)	0.688	2 (1.2)	1 (1.6)	0.820
Physical inactivity, *n* (%)	48 (22.4)	34 (23.6)	0.794	103 (62.1)	40 (63.5)	0.840
*Variables assessed during the telephone interview (May–September 2020)*						
Living alone, *n* (%)	69 (32.2)	54 (37.5)	0.304	109 (65.7)	40 (63.5)	0.758
Location where most time spent (since March), *n* (%)						
Home/with relatives	209 (100.0)	141 (99.3)	0.224	155 (95.1)	58 (92.1)	0.381
Senior, service, nursing housing/hospital/rehab	0 (0.0)	1 (0.7)		8 (4.9)	5 (7.9)	
Frequency of going out (since March), *n* (%)						
Everyday	190 (89.2)	126 (88.7)	0.161	123 (74.6)	42 (66.7)	0.356
≥ 1 per week	22 (10.3)	12 (8.5)		30 (18.2)	13 (20.6)	
< 1 per week/ never	1 (0.5)	4 (2.8)		12 (7.3)	8 (12.7)	
Use of public transport (since March), *n* (%)						
≥ 1 per week	26 (12.2)	26 (18.2)	0.270	18 (11.0)	6 (9.5)	0.520
2–3 times per month	21 (9.9)	15 (10.5)		15 (9.2)	9 (14.3)	
< 2 times per month/never	166 (77.9)	102 (71.3)		131 (79.9)	48 (73.2)	

Physical inactivity defined according to current guidelines’ definition of moderate-to-vigorous physical activity levels (i.e., < 150 min/week)

*COPD* chronic obstructive pulmonary disease

*Chi^2^ test

**Table 6:** Associations of poor muscle strength, mobility limitation, and physical activity levels with presence of COVID-19-like symptoms (0 vs 1 +) in the physical activity assessment sub-sample by age groups. Results from logistic regression models after mutually adjusting by all exposures (*n* = 587)

	Total sub-sample		Age (< 70 years)		Age (≥ 80 years)	
	OR (95% CI)	*p* value	OR (95% CI)	*p* value	OR (95% CI)	*p* value
Poor muscle strength						
No	Ref.	Ref.	Ref.	Ref.	Ref.	Ref.
Yes	**1.5 (1.0–2.2)**	**0.066**	1.1 (0.7–1.9)	0.625	**3.2 (1.4–7.1)**	**0.004**
Mobility limitation						
No	Ref.	Ref.	Ref.	Ref.	Ref.	Ref.
Yes	0.7 (0.4–1.5)	0.389	2.0 (0.5–8.2)	0.343	0.6 (0.3–1.4)	0.264
Physical activity						
Active	Ref.	Ref.	Ref.	Ref.	Ref.	Ref.
Inactive	1.0 (0.6–1.5)	0.962	1.0 (0.6–1.7)	0.942	0.9 (0.4–1.8)	0.705

Muscle strength, mobility limitation, and physical activity were analyzed together, further adjusting by age sex, education level, living alone, location where most time spent, frequency of going out, use of public transport, number of chronic cardiovascular, neuropsychiatric, and musculoskeletal diseases, COPD, asthma, and other respiratory diseases. Poor muscle strength defined as a chair stand test time below the median of the distribution (i.e., ≥ 11 s). Mobility limitation defined according to a previously suggested clinical cut-off for walking speed (i.e., < 0.8 m/s). Physical inactivity defined according to current guidelines’ definition of moderate-to-vigorous physical activity levels (i.e., < 150 min/week). Interaction with age: poor muscle strength (yes vs no) # age (≥ 80 years vs < 70 years): OR = 2.25; *p* = 0.077. Mobility limitation (yes vs no) # age (≥ 80 years vs < 70 years): OR = 0.26; *p* = 0.094. Physical activity (inactive vs active) # age (≥ 80 years vs < 70 years): OR = 0.93; *p* = 0.866

**Table 7:** Associations of poor muscle strength, mobility limitation, and physical activity with number of COVID-19-like symptoms in the total sample and by age groups. Results from linear regression models

	Total sample		Age (< 70 years)		Age (≥ 80 years)	
	*β* (95% CI)	*p* value	*β* (95% CI)	*p* value	*β* (95% CI)	*p* value
Poor muscle strength (*N* = 904)						
No	Ref.	Ref.	Ref.	Ref.	Ref.	Ref.
Yes	0.04 (− 0.2 to 0.3)	0.708	− 0.13 (− 0.5 to 0.2)	0.426	**0.28 (0.04 to 0.5)**	**0.024**
Mobility limitation (*N* = 904)						
No	Ref.	Ref.	Ref.	Ref.	Ref.	Ref.
Yes	− 0.10 (− 0.4 to 0.2)	0.484	0.11 (− 0.7 to 0.9)	0.799	− 0.16 (− 0.4 to 0.8)	0.199
Physical activity (*n* = 587)						
Active	Ref.	Ref.	Ref.	Ref.	Ref.	Ref.
Inactive	0.09 (− 0.2 to 0.4)	0.502	0.20 (− 0.2 to 0.6)	0.320	− 0.07 (− 0.4 to 0.3)	0.663

Models adjusted by age, sex, education level, living alone, location where most time spent, frequency of going out, use of public transport, number of chronic cardiovascular, neuropsychiatric, and musculoskeletal diseases, COPD, asthma, and other respiratory diseases. Poor muscle strength defined as a chair stand test time below the median of the distribution (i.e., ≥ 11 s). Mobility limitation defined according to a previously suggested clinical cut-off for walking speed (i.e., < 0.8 m/s). Physical inactivity defined according to current guidelines’ definition of moderate-to-vigorous physical activity levels (i.e., < 150 min/week). Interaction with age: Poor muscle strength (yes vs no) # age (≥ 80 years vs < 70 years): *β* = 0.32; *p* = 0.130. Mobility limitation (yes vs no) # age (≥ 80 years vs < 70 years): *β* = − 0.44; *p* = 0.232. Physical activity (inactive vs active) # age (≥ 80 years vs < 70 years): β = − 0.26; *p* = 0.313

**Table 8:** Associations of poor muscle strength, mobility limitation, and physical activity levels with presence of COVID-19-like symptoms (0–1 vs 2 +) in the total sample and by age groups. Results from logistic regression models

	Total sample		Age (< 70 years)		Age (≥ 80 years)	
	OR (95% CI)	*p* value	OR (95% CI)	*p* value	OR (95% CI)	*p* value
Poor muscle strength (*N* = 904)						
No	Ref.	Ref.	Ref.	Ref.	Ref.	Ref.
Yes	1.04 (0.7–1.5)	0.851	0.79 (0.5–1.3)	0.344	2.1 (0.9–4.6)	0.077
Mobility limitation (*N* = 904)						
No	Ref.	Ref.	Ref.	Ref.	Ref.	Ref.
Yes	0.70 (0.4–1.3)	0.249	0.83 (0.3–2.7)	0.760	0.64 (0.3–1.3)	0.233
Physical activity (*n* = 587)						
Active	Ref.	Ref.	Ref.	Ref.	Ref.	Ref.
Inactive	1.1 (0.7–1.8)	0.765	1.43 (0.8–2.7)	0.267	0.54 (0.2–1.4)	0.202

Models adjusted by age, sex, education level, living alone, location where most time spent, frequency of going out, use of public transport, number of chronic cardiovascular, neuropsychiatric and musculoskeletal diseases, COPD, asthma, and other respiratory diseases, Poor muscle strength defined as a chair stand test time below the median of the distribution (i.e., ≥ 11 s). Mobility limitation defined according to a previously suggested clinical cut-off for walking speed (i.e., < 0.8 m/s). Physical inactivity defined according to current guidelines’ definition of moderate-to-vigorous physical activity levels (i.e., < 150 min/week). Interaction with age: poor muscle strength (yes vs no) # age (≥ 80 years vs < 70 years): OR = 2.35; *p* = 0.063. Mobility limitation (yes vs no) # age (≥ 80 years vs < 70 years): OR = 0.72; *p* = 0.625. Physical activity (inactive vs active) # age (≥ 80 years vs < 70 years): OR = 0.51; *p* = 0.199
